# Real‐World Safety and Efficacy of Biosimilar Trastuzumab Emtansine (T‐DM1) in HER2‐Positive Breast Cancer: A Prospective Single‐Institution Study From India

**DOI:** 10.1155/ijbc/8863217

**Published:** 2026-04-20

**Authors:** Divya Gandrala, Senthil J. Rajappa, Krishnamohan Mallavarapu, A. Santa, B. Pavan Kumar, Rakesh Pinninti, Pallavi S. Ladda, Nikhil Pathi, Sanath Kandem, Rohan Kamlesh Tewani, Vipulkumar Thummar, Priya Mehta

**Affiliations:** ^1^ Department of Medical Oncology, Basavatarakam Indo American Cancer Hospital and Research Institute, Hyderabad, India, induscancer.com; ^2^ Medical Affairs, Zydus Lifesciences Ltd, Ahmedabad, India

**Keywords:** HER2-positive breast cancer, overall survival, targeted therapy, T-DM1, progression-free survival

## Abstract

**Background:**

Trastuzumab emtansine (T‐DM1) represents an advancement in HER2‐targeted therapy, offering a unique combination of trastuzumab′s specificity and the cytotoxic potency of maytansine. It demonstrates therapeutic efficacy across the spectrum of breast cancer, including both early‐stage and advanced disease. With the emergence of biosimilar formulations, there is a growing need to generate real‐world evidence supporting their clinical performance. This study investigates the effectiveness and safety of a T‐DM1 biosimilar in routine oncology practice across adjuvant and metastatic HER2‐positive breast cancer populations.

**Methods:**

In this prospective, single‐institute study conducted at a tertiary oncology center in India, 116 patients with HER2‐positive breast cancer (51 adjuvant and 65 metastatic) received T‐DM1 biosimilar (Ujvira) between June 2021 and October 2023. Outcomes assessed included overall response rate (ORR), progression‐free survival (PFS), overall survival (OS), and treatment‐emergent adverse events (AEs), graded using CTCAE v5.0.

**Results:**

In the metastatic cohort, ORR was 63.4%, median PFS was 8.0 months (95% CI: 5.8–10.2), and estimated median OS was 18.0 months (95% CI: 16.1–19.9). Clinical benefit rate was 75%. In the adjuvant cohort, 62.7% completed the planned 14 cycles, and treatment was well tolerated. Grade ≥ 3 AEs occurred in 6.9% of all patients, with fatigue and thrombocytopenia being the most common AEs. No unexpected safety signals were observed.

**Conclusion:**

T‐DM1 biosimilar showed outcomes consistent with those reported for the originator in real‐world settings, supporting its broader adoption in both early and advanced HER2‐positive breast cancer. Its affordability may improve access and outcomes in resource‐limited settings.

## 1. Introduction

Antibody‐drug conjugates (ADCs) have emerged as a promising class of targeted cancer therapies in oncology, particularly in the treatment of HER2‐positive breast cancer, by combining the tumor‐specific binding capacity of monoclonal antibodies with the potent cytotoxic activity of chemotherapeutic agents. Among these, trastuzumab emtansine (T‐DM1) is a well‐established ADC, combining the HER2‐targeting action of trastuzumab with the microtubule‐inhibitory effect of the cytotoxic agent maytansine (DM1). This conjugate retains trastuzumab′s HER2‐binding specificity while enabling targeted intracellular delivery of DM1, thereby exerting a dual mechanism of action [1]. T‐DM1 has demonstrated significant benefit in both metastatic and early‐stage HER2‐positive breast cancer, as established by pivotal trials such as EMILIA and KATHERINE. Given that HER2‐positive tumors account for approximately 15%–20% of all breast cancers and are typically associated with aggressive disease behavior, the therapeutic role of T‐DM1 is of considerable importance. However, these trials were predominantly conducted in Western populations. Moreover, the cost of T‐DM1 remains a substantial barrier to access in LMICs, often limiting the implementation of guideline‐recommended therapies for a substantial proportion of patients, despite well‐established clinical efficacy. [2]

In May 2021, the world′s first biosimilar of T‐DM1 was made available in India after regulatory approval by the Drugs Controller General of India (DCGI) [3, 4]. Although international guidelines such as those from the National Comprehensive Cancer Network (NCCN) recommend T‐DM1 for residual disease and metastatic settings, such recommendations are grounded in trial settings that may not reflect the reality of clinical practice in LMICs [5]. Biosimilar T‐DM1 has predominantly been used in Indian clinical practice as a second‐line treatment in the metastatic setting, particularly for patients who have progressed on trastuzumab‐based regimens, consistent with evidence from the EMILIA trial [6]. More recently, its adoption in the adjuvant setting is increasing for patients with residual invasive HER2‐positive disease following neoadjuvant therapy, reflecting the paradigm shift introduced by the KATHERINE trial [7]. Yet, data on the real‐world effectiveness, safety, and tolerability of biosimilar T‐DM1 in routine Indian clinical settings are limited, especially for patients with both early and advanced HER2‐positive breast cancer.

Despite the well‐established efficacy of T‐DM1 in HER2‐positive breast cancer, access remains limited in low‐ and middle‐income countries (LMICs) due to its high cost and reliance on data derived predominantly from Western populations. Real‐world evidence evaluating the safety and effectiveness of biosimilar T‐DM1, particularly in diverse patient populations and across both adjuvant and metastatic settings, is currently scarce. To address this gap, we conducted a prospective real‐world study involving HER2‐positive breast cancer patients treated with T‐DM1 biosimilar at a tertiary cancer center in India.

## 2. Materials and Methods

### 2.1. Study Design

This was a prospective, real‐world study conducted at Basavatarakam Indo‐American Cancer Hospital and Research Institute, a tertiary oncology center in Hyderabad, India. The study included patients with HER2‐positive breast cancer across adjuvant, advanced, and metastatic stages. It was conducted over a 28‐month period, from June 1, 2021, to October 20, 2023.

### 2.2. Study Objective

This study is aimed at evaluate the efficacy and safety of biosimilar T‐DM1 in routine clinical practice among the patients with early and advanced HER2‐positive breast cancer.

### 2.3. Study Population

A total of 116 patients with HER2‐positive breast cancer who received at least one dose of T‐DM1 biosimilar (UJVIRA, Zydus Lifesciences Ltd) during the study period were prospectively enrolled. HER2‐positivity was established in accordance with institutional protocols, based on either immunohistochemistry (IHC 3+) or confirmed amplification by fluorescence in situ hybridization (FISH). Biosimilar T‐DM1 (Ujvira) is procured by patients either through direct out‐of‐pocket purchase or via insurance/financial assistance schemes. No sponsor‐funded drug support or free‐of‐cost supply was involved in this study.

### 2.4. Sample Size Calculation

As this was a real‐world, prospective cohort study, no a priori formal sample size calculation was performed. All consecutive eligible patients who received at least one dose of biosimilar T‐DM1 during the study period were included to maximize representativeness and reduce selection bias. Follow‐up data were available for all enrolled patients, and there was no loss to follow‐up during the study period. Missing laboratory or imaging parameters were infrequent (< 5%) and were handled using complete‐case analysis, consistent with real‐world study methodology.

### 2.5. Baseline Examination and Study Follow‐Up

All patients underwent a comprehensive baseline assessment prior to initiation of therapy, including clinical history, physical examination, hematological investigations (complete blood counts, liver, and renal function tests), and cardiac assessment via echocardiography to evaluate left ventricular ejection fraction (LVEF). Radiological imaging with either contrast‐enhanced computed tomography (CT) or positron emission tomography—computed tomography (PET‐CT) was performed to assess disease burden and establish measurable lesions for response evaluation using RECIST v1.1 criteria. All scans were interpreted by board‐certified radiologists in our institution, and treating oncologists assessed response based on these radiology reports; no independent central radiologic review was performed. Patients were followed during treatment with clinical assessments and laboratory monitoring conducted before each treatment cycle. Imaging was repeated every three to four cycles, or earlier if clinically indicated, to evaluate treatment response. Adverse events (AEs) were documented at each visit and graded according to Common Terminology Criteria for Adverse Events (CTCAE) v5.0.

### 2.6. Effectiveness and Safety Assessment

Effectiveness was assessed based on objective response rate (ORR), clinical benefit rate (CBR), progression‐free survival (PFS), and overall survival (OS). ORR was defined as the proportion of patients achieving complete or partial response, whereas CBR included those with complete response, partial response, or stable disease. PFS was calculated from the date of the first dose of T‐DM1 to the date of documented disease progression or death, and OS was measured from treatment initiation to death or last follow‐up. Safety assessment included continuous monitoring and documentation of treatment‐emergent AEs at each visit. Radiological responses were assessed by the treating medical oncologists based on formal reports issued by board‐certified radiologists at our institution. No independent central radiologic review was performed.

### 2.7. Statistical Analysis

Statistical analysis was performed using SPSS Version 27.0. Descriptive statistics were used to summarize baseline characteristics. PFS and OS were estimated using the Kaplan–Meier method, with medians and 95% confidence intervals (CIs) reported. ORR and CBR were calculated based on radiological assessments per RECIST v1.1. Subgroup analyses of ORR were performed using chi‐square or Fisher′s exact test, with a *p* value < 0.05 considered statistically significant. Median follow‐up was estimated using the reverse Kaplan–Meier method. In this approach, the event indicator is reversed so that deaths are treated as censored and patients who remain alive at the last contact are considered “events;” this yields the median observation time to censoring, representing the median follow‐up for participants.

### 2.8. Ethical Considerations

The study was approved by the Ethics Committee of Basavatarakam Indo‐American Cancer hospital and research institute, Hyderabad with reference code IEC/2025/AC/40.

## 3. Results

### 3.1. Baseline Characteristics—Adjuvant Disease

The median age of patients in the adjuvant cohort was 54 years (interquartile range [IQR]: 47–58 years). All 51 patients received T‐DM1 biosimilar as part of adjuvant therapy and were followed for a median duration of 14 months. Among these, 30 (58.8%) were hormone receptor‐positive (ER and/or PR), whereas 21 (41.2%) were hormone receptor‐negative. With respect to neoadjuvant HER2‐targeted therapy, the majority, 44 (86.3%) had received trastuzumab alone. Baseline demographic and treatment characteristics of the adjuvant cohort are detailed in Table 1.

**Table 1:** Demographic and clinical characteristics of the patients—adjuvant stage disease.

Characteristic	Adjuvant (*n* = 51)
Median age (median‐ year)	54 (IQR 47–58)
Hormone receptor status	
ER/PR positive *n* (%)	30 (58.8%)
ER/PR negative *n* (%)	21 (41.2%)
Prior use of anthracyclines—no of patients (%)	30 (58.8%)
Neoadjuvant Her2 targeted therapy—no of patients (%)	
Trastuzumab alone *n* (%)	44 (86.3%)
Trastuzumab + pertuzumab*n* (%)	7 (13.7%)

### 3.2. Baseline Characteristics—Advanced Disease

In the advanced‐stage cohort comprising 65 patients, 34 patients (52.3%) had an ECOG performance status of ≥ 2 at baseline, indicating a moderately impaired functional status. Visceral metastases were present in 45 patients (69.2%), reflecting a high burden of systemic disease. Regarding hormone receptor status, 35 patients (53.8%) were ER/PR negative, whereas the remaining 46.2% were hormone receptor positive. Prior exposure to anthracyclines was documented in 20 patients (30.8%), and 9 patients (13.8%) had received more than one prior chemotherapy regimen before initiating T‐DM1. The detailed characteristics of the advanced cohort are summarized in Table 2.

**Table 2:** Demographic and clinical characteristics of the patients—advanced stage disease.

Characteristic	Advanced (*n* = 65)
Median age (median (IQR year)	53 (IQR 46–61)
ECOG PS—n (%)	
0–1	31 (47.7%)
≥ 2	34 (52.3%)
Site of metastases—no (%)	
Visceral	45 (69.2%)
Nonvisceral	20 (30.8%)
Hormone receptor status—no (%)	
ER/PR positive	30 (46.2%)
ER/PR negative	35 (53.8%)
Prior systemic therapy—no (%)	
Anthracyclines	20 (30.8%)
Other chemo	45 (69.2%)
Prior chemo regimens—no (%)	
1	55 (84.6%)
> 1	9 (13.8%)

### 3.3. Objective Response and Clinical Benefit in Metastatic Patients

**Figure 1:**
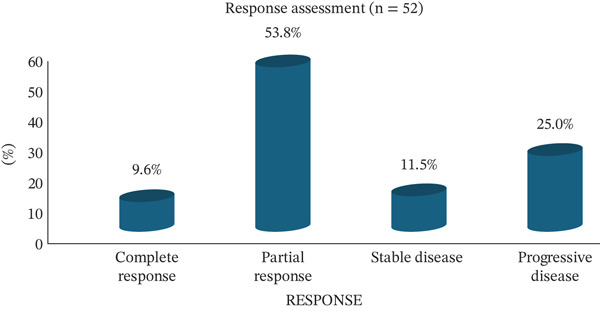
Radiological response and clinical benefit in metastatic patients.

Out of 65 patients with metastatic HER2‐positive breast cancer who received T‐DM1 biosimilar, 52 were evaluable for radiological response. At a median follow‐up of 9 months, the ORR was 63.4%, with 9.6% achieving complete response and 53.8% partial response. An additional 11.5% of patients achieved stable disease, yielding a CBR of 75%. The median duration of response was 5 months (IQR: 3–9 months), and 25% of patients experienced progressive disease despite therapy (Figure 1).

Subgroup analysis was performed among the response‐evaluable population (*n* = 52). ORR was 70.8% (17/24) among hormone receptor–positive patients and 57.1% (16/28) among hormone receptor–negative patients (*p* = 0.39). ORR was 53.3% (8/15) in patients with CNS metastases and 67.6% (25/37) in those without CNS involvement (*p* = 0.36). Patients with visceral metastases demonstrated an ORR of 68.6% (24/35), compared with 52.9% (9/17) among those without visceral involvement (*p* = 0.36). A lower response rate was observed in patients who had received more than one prior chemotherapy regimen (44.4% vs. 67.4%; *p* = 0.26) (Table 3).

**Table 3:** Response based on baseline characteristics.

Baseline characteristic	Responders *n*/*N* (%)	*p*value
Hormone receptor status		
ER/PR positive (*n* = 24)	17/24 (70.8%)	0.39
ER/PR negative (*n* = 28)	16/28 (57.1%)	
CNS metastases		
Yes (*n* = 15)	8/15 (53.3%)	0.36
No (*n* = 37)	25/37 (67.6%)	
Visceral metastases		
Yes (*n* = 35)	24/35 (68.6%)	0.36
No (*n* = 17)	9/17 (52.9%)	
Prior chemotherapy regimens		
1 prior line (*n* = 43)	29/43 (67.4%)	0.26
> 1 prior lines (*n* = 9)	4/9 (44.4%)	

### 3.4. PFS and OS

PFS was assessed in the metastatic breast cancer cohort treated with T‐DM1 biosimilar. At a median follow‐up of 9 months, the median progression‐free survival (mPFS) was 8.0 months (95% CI: 5.8–10.2 months). The mean PFS was 11.0 months (95% CI: 8.5–13.5 months). The OS was analyzed for patients receiving T‐DM1 biosimilar in the metastatic setting. At a median follow‐up of 9 months, the median overall survival (mOS) was 18.0 months (95% CI: 16.1–19.9 months). The estimated mean OS was 17.9 months (95% CI: 15.1–20.8 months). (Figure 2).

Figure 2(a) Kaplan–Meier for median progression‐free survival. (b) Kaplan–Meier for median overall survival.

**Figure:**
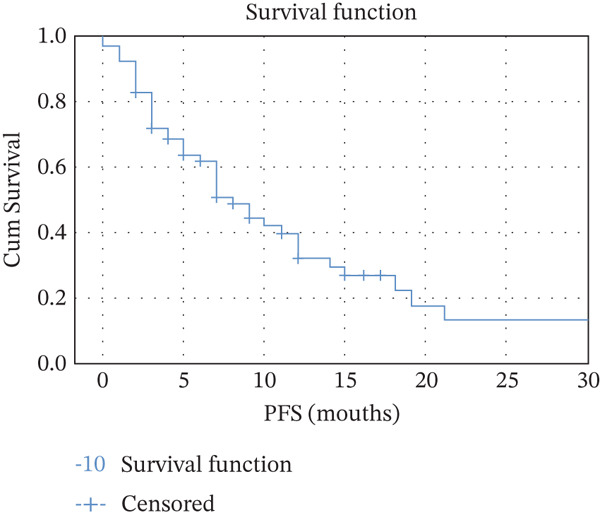
(a)

**Figure:**
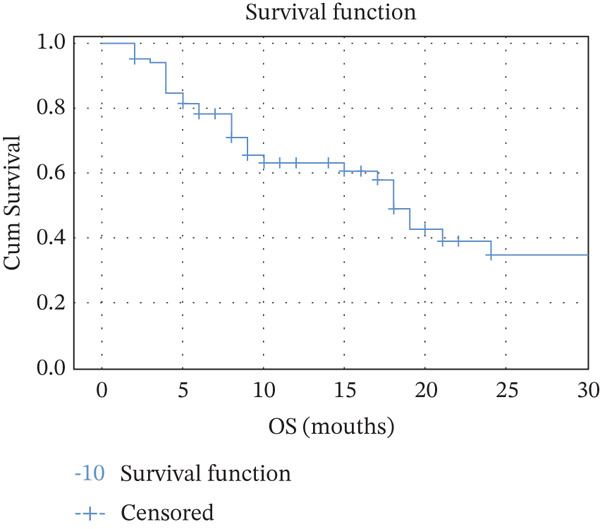
(b)

### 3.5. Safety Assessment in Both Adjuvant and Advanced Disease

Overall, 60.3% of patients (*n* = 70) experienced at least one treatment‐emergent AE, with 6.9% (*n* = 8) developing grade ≥ 3 AEs. Fatigue (36.2%) and thrombocytopenia (19.8%) were the most commonly reported toxicities, though most were low grade. Diarrhea (5.2%), nausea (10.3%), and vomiting (6.9%) were also observed but did not lead to treatment discontinuation. Grade ≥ 3 thrombocytopenia was seen in 2.6% of patients, whereas elevated ALT and AST occurred in 1.7% each. Eight patients (6.9%) required admission for supportive care, and 21.5% (*n* = 25) required a dose reduction. No new safety signals were observed, and the overall toxicity profile was consistent with the known effects of T‐DM1. (Table 4).

**Table 4:** Adverse events in overall population (*n* = 116).

Adverse event	Any grade, *n* (%)	95% CI (any grade)	Grade ≥ 3, *n* (%)	95% CI (grade ≥ 3)
Any event	70 (60.3%)	50.9%–69.2%	8 (6.9%)	3.1%–13.1%
Diarrhea	6 (5.2%)	2.0%–10.9%	1 (0.9%)	0.0%–4.7%
Vomiting	8 (6.9%)	3.1%–13.1%	0	—
Fatigue	42 (36.2%)	27.6%–45.6%	0	—
Nausea	12 (10.34%)	5.6%–17.3%	0	—
Anemia	10 (8.62%)	4.3%–15.3%	0	—
Neutropenia	10 (8.62%)	4.3%–15.3%	0	—
Thrombocytopenia	23 (19.8%)	13.2%–28.1%	3 (2.6%)	0.5%–7.4%
Elevated ALT	6 (5.2%)	2.0%–10.9%	2 (1.7%)	0.2% ‐6.0%
Elevated AST	6 (5.2%)	2.0%–10.9%	2 (1.7%)	0.2%–6.0%
Admitted for supportive care	8 (6.9%)	3.1%–13.1%	—	—
Dose reduction	25 (21.5%)	14.7%–29.9%	—	—

### 3.6. Comparison of AEs Between Adjuvant and Advanced Treatment Groups

AEs were graded using the (CTCAE) Version 5.0 [8]. The incidence of any‐grade AEs was significantly higher in the advanced group (69.2%, 95% CI: 57.2%–80.0%) compared with the adjuvant group (49.0%, 95% CI: 35.4%–62.7%) (*p* = 0.02). Grade ≥ 3 events occurred infrequently and were comparable between the two groups (7.7% vs. 5.9%). No statistically significant differences were observed in the rates of specific AEs, including fatigue (43.1% vs. 27.4%; *p* = 0.08), nausea (6.2% vs. 15.7%; *p* = 0.09), or thrombocytopenia (17.0% vs. 25.5%; *p* = 0.25). The frequency of dose reductions (21.5% vs. 21.6%; *p* = 0.99) and admissions for supportive care (9.2% vs. 3.9%; *p* = 0.26) was similar across both cohorts. (Table 5).

**Table 5:** Comparison of Adverse events in adjuvant and advanced group.

	Adjuvant (*n* = 51)				Advanced (*n* = 65)				
Adverse event	Any grade *N*(%)	Grade ≥ 3	95% CI (any grade)	95% CI (grade ≥ 3)	Any grade	Grade ≥ 3	95% CI (any grade)	95% CI (grade ≥)	*p* *value*
Any event	25 (49%)	3 (5.9%)	35.0%–63.2%	1.2% ‐16.2%	45 (69.2%)	5 (7.7%)	56.6%–79.9%	2.5%–17.0%	0.02
Diarrhea	2 (3.9%)	1 (1.9%)	0.5%–13.5%	0.0% ‐10.4%	4 (6.2%)	0	1.7%–14.9%		0.59
Vomiting	4 (7.8%)	0	2.2%–18.9%	—	4 (6.2%)	0	1.7%–14.9%		0.72
Fatigue	14 (27.4%)	0	16.1%–41.9%	—	28 (43.1%)	0	31.0%–55.8%		0.08
Nausea	8 (15.7%)	0	7.0%–28.6%	—	4 (6.2%)	0	1.7%–14.9%		0.09
Anemia	4 (7.8%)	0	2.2%–18.9%	—	6 (9.2%)	0	3.5%–19.0%		0.79
Neutropenia	4 (7.8%)	0	2.2%–18.9%	—	6 (9.2%)	0	3.5%–19.0%		0.79
Thrombocytopenia	13 (25.5%)	1 (1.9%)	14.6%–39.6%	0.0% ‐10.4%	11 (17%)	3 (4.6%)	8.8%–28.3%	1.0%–12.9%	0.25
Elevated ALT	2 (3.9%)	1 (1.9%)	0.5%–13.5%	0.0% ‐10.4%	4 (6.2%)	1 (1.5%)	1.7%–14.9%	0.0%–8.2%	0.59
Elevated AST	2 (3.9%)	1 (1.9%)	0.5%–13.5%	0.0% ‐10.4%	4 (6.2%)	1 (1.5%)	1.7%–14.9%	0.0%–8.2%	0.59
Admitted for supportive care	2 (3.9%)		0.5%–13.5%	—	6 (9.2%)		3.5%–19.0%		0.26
Dose reduction	11 (21.6%)		11.4%–35.5%	—	14 (21.5%)		12.3%–33.5%		0.99

## 4. Discussion

In the metastatic cohort treated with biosimilar T‐DM1, the ORR was 63.4%, with a mPFS of 8 months and a median OS of 18 months at the time of analysis. The Phase III EMILIA trial [9] (second‐line T‐DM1 vs. lapatinib + capecitabine) demonstrated an ORR of 43.6%, mPFS of 9.6 months, and median OS of 30.9 months in the T‐DM1 arm. In contrast, the later‐line TH3RESA trial [10] (patients treated after ≥ 2 prior HER2 therapies) reported a lower mPFS and an ORR around 30%, consistent with a more heavily pretreated population, with a median OS of 22.7 months on T‐DM1. Our real‐world ORR and PFS fall between EMILIA and TH3RESA, likely reflecting real‐world patient heterogeneity and the fact that most patients received T‐DM1 as second‐line therapy. A single‐center study from the Royal Marsden [11] reported an ORR of 64% with T‐DM1, alongside a mPFS of 8.7 months. A recent study by Patel et al. conducted using the same Ujvira biosimilar (*n* = 30 metastatic; no patient overlap despite shared investigators), reported CR 66.6%, PR 33.3%, and a mPFS of 4 months in a lower‐risk cohort (13% ECOG ≥ 2 vs. our 52%) [12]. The high response rate in that study was attributed to patient selection (many had only one line of prior therapy), reinforcing that T‐DM1 achieves its best efficacy when used earlier in the metastatic course. Overall, the tumor response and disease control observed with T‐DM1 biosimilar in our study mirror the efficacy of the originator T‐DM1 in clinical trials, confirming that our patients derived substantial benefit from this therapy. Preliminary institutional data from the same center were presented at ASCO 2024 as an interim retrospective audit of early biosimilar T‐DM1 use; the present manuscript builds on this by reporting a prospectively followed real‐world cohort with predefined objectives, standardized response assessment (RECIST v1.1), CTCAE v5.0 toxicity grading, and updated outcome reporting.

The Royal Marsden experience [11] also reported a median OS of 20.4 months with T‐DM1. In comparison, the shorter OS observed in our study may be attributed to several adverse prognostic factors within our patient population, including a substantial proportion presenting with visceral crisis or discontinuing treatment early for nonmedical reasons. Differences in OS across studies may also reflect variations in baseline performance status, burden of visceral disease, prior lines of therapy, and access to subsequent treatment. Additionally, the lower survival rates may also reflect the fact that many patients did not receive subsequent lines of therapy due to poor performance status.

The safety profile of biosimilar T‐DM1 observed in our study closely matches the known toxicity spectrum of T‐DM1 from clinical trials. Overall, treatment was well tolerated, with fewer high‐grade AEs than typically seen with conventional chemotherapy combinations. In the EMILIA trial, 41% of patients on T‐DM1 experienced grade ≥ 3 AEs, significantly lower than the 57% with lapatinib + capecitabine. In our study, serious toxicities were likewise infrequent; only 7.7% of advanced‐stage patients discontinued T‐DM1 due to intolerance (and no grade ≥ 3 events were seen in the adjuvant subgroup). We observed no new or unexpected AEs with the biosimilar. AEs were prospectively documented and graded according to CTCAE v5.0 during routine follow‐up, supporting the robustness of safety reporting in this real‐world cohort.

These findings must be interpreted within the real‐world context of a LMIC, where access to high‐cost oncology drugs is limited. Prior to the availability of a biosimilar, the prohibitive cost of originator T‐DM1 (Kadcyla) in India restricted its use to patients with financial means or exceptional support. The introduction of biosimilar T‐DM1, priced 80% lower, significantly improved affordability and access. Following biosimilar availability, institutional utilization increased more than threefold compared with the prebiosimilar period, suggesting improved accessibility. At our center, this enabled many patients who would otherwise discontinue HER2‐directed therapy post first‐line treatment to receive second‐line or adjuvant T‐DM1, achieving outcomes comparable with those reported in high‐income settings. The availability of biosimilar T‐DM1 has critical implications for healthcare equity in LMICs. In India, where HER2‐positive breast cancer often affects younger women and exhibits aggressive behavior, improved access to T‐DM1 may translate into better survival and reduced recurrence.

A substantial number of patients in our study received T‐DM1 in the adjuvant setting, following neoadjuvant chemotherapy and surgery [13]. This practice is driven by the landmark KATHERINE trial, which established adjuvant T‐DM1 as the standard of care for patients with residual invasive disease after neoadjuvant HER2‐directed therapy. In KATHERINE, switching to T‐DM1 (instead of continuing trastuzumab) led to a 50% reduction in the risk of invasive disease recurrence. At 3 years follow‐up, invasive disease‐free survival (IDFS) was 88.3% in the T‐DM1 arm, compared with 77.0% with trastuzumab alone, an absolute improvement of over 11%, which translated into improved OS with longer follow‐up [14]. These results have made adjuvant T‐DM1 the therapy of choice for HER2‐positive patients who do not achieve a pathological complete response to neoadjuvant treatment. Our real‐world experience corroborates the feasibility and benefit of T‐DM1 in the adjuvant context. Among the 51 early‐stage patients in our cohort, 62.7% completed the planned 14 cycles of T‐DM1, and an additional 27.5% were still on therapy at the time of analysis (i.e., treatment ongoing). This completion rate is quite encouraging given that adjuvant therapy is often stopped early in practice due to toxicity or patient preference. The most common side effect was fatigue, as in the metastatic setting, but this was manageable and did not lead to therapy discontinuation. These findings support the feasibility of delivering adjuvant T‐DM1 in routine clinical practice within resource‐constrained settings.

A recent retrospective single‐center study of 30 Indian patients treated with biosimilar T‐DM1 reported high response rates and a median time‐to‐treatment failure of 13 months. However, that analysis was limited by its small sample and retrospective design [15]. Our prospective study enrolled 116 patients, including both adjuvant and metastatic cohorts, and provides broader real‐world evidence on efficacy and safety. In addition, no patients from the earlier retrospective review were included in the present dataset, ensuring independence of the analyses.

In India, the originator T‐DM1 (Kadcyla) historically cost between INR 1.2–1.8 lakhs per cycle, placing it beyond reach for most patients in the public and even private sectors [16]. The biosimilar formulation is available at approximately 70%–80% lower acquisition cost, substantially reducing the financial burden on patients and enabling wider use across both adjuvant and metastatic settings [15]. Although our study did not perform a formal pharmacoeconomic analysis, the marked reduction in cost directly contributed to increased uptake of second‐line HER2‐directed therapy at our center. In LMICs, health‐economic evaluations consistently demonstrate that reduced drug acquisition costs are a critical factor influencing the initiation and continuation of treatment for advanced breast cancer. Therefore, improving access to guideline‐recommended HER2‐targeted therapies is expected to deliver significant clinical benefits at the population level, particularly in settings where treatment discontinuation due to financial constraints is common. This is supported by evidence showing that biosimilar T‐DM1 formulations, through substantial cost reductions, enhance treatment accessibility and adherence, ultimately improving outcomes in HER2‐positive breast cancer patients in these regions [16, 17].

It is a single‐institution analysis, and although prospective in design, it lacks a comparator arm. The follow‐up period is relatively short, particularly for the metastatic cohort, limiting the interpretation of long‐term OS outcomes. At the data cutoff, the median follow‐up (reverse Kaplan–Meier) was 9 months. Because follow‐up is shorter than the estimated median OS of 18 months, the OS curve remains immature and should be interpreted cautiously. Additionally, although AEs were rigorously documented, quality‐of‐life measures and patient‐reported outcomes were not included, an area that warrants exploration in future studies. It provides critical evidence that biosimilar T‐DM1 retains clinical efficacy and safety comparable with its originator, with additional value through improved affordability and access. Our findings support its adoption across HER2‐positive breast cancer indications, reinforcing its role as a standard‐of‐care agent in both curative and palliative intent settings.

## 5. Conclusion

This real‐world study demonstrates that biosimilar T‐DM1 offers comparable efficacy and safety to the innovator product in both adjuvant and metastatic HER2‐positive breast cancer. Treatment was well tolerated, with manageable toxicity and no new safety concerns. Importantly, the availability of a cost‐effective biosimilar has improved access to standard‐of‐care therapy in India. These findings support the integration of biosimilar T‐DM1 into clinical practice across diverse treatment settings.

## Funding

No funding was received for this manuscript.

## Conflicts of Interest

The authors declare no conflicts of interest.

## Data Availability

The data that support the findings of this study are available on request from the corresponding author. The data are not publicly available due to privacy or ethical restrictions.
